# Molecular Mechanisms of Protein Aggregation in ALS-FTD: Focus on TDP-43 and Cellular Protective Responses

**DOI:** 10.3390/cells14100680

**Published:** 2025-05-08

**Authors:** Enza Maria Verde, Valentina Secco, Andrea Ghezzi, Jessica Mandrioli, Serena Carra

**Affiliations:** Department of Biomedical, Metabolic and Neural Sciences, University of Modena and Reggio Emilia, 41125 Modena, Italy; v.enzamaria96@gmail.com (E.M.V.); valentina.secco@unimore.it (V.S.); andreaghezzi140495@gmail.com (A.G.); jmandrio@unimore.it (J.M.)

**Keywords:** ALS-FTD, TDP-43, protein aggregation, stress granules, post-translational modifications

## Abstract

Amyotrophic Lateral Sclerosis (ALS) and Frontotemporal Dementia (FTD) are two neurodegenerative disorders that share common genes and pathomechanisms and are referred to as the ALS-FTD spectrum. A hallmark of ALS-FTD pathology is the abnormal aggregation of proteins, including Cu/Zn superoxide dismutase (SOD1), transactive response DNA-binding protein 43 (TDP-43), fused in sarcoma/translocated in liposarcoma (FUS/TLS), and dipeptide repeat proteins resulting from C9orf72 hexanucleotide expansions. Genetic mutations linked to ALS-FTD disrupt protein stability, phase separation, and interaction networks, promoting misfolding and insolubility. This review explores the molecular mechanisms underlying protein aggregation in ALS-FTD, with a particular focus on TDP-43, as it represents the main aggregated species inside pathological inclusions and can also aggregate in its wild-type form. Moreover, this review describes the protective mechanisms activated by the cells to prevent protein aggregation, including molecular chaperones and post-translational modifications (PTMs). Understanding these regulatory pathways could offer new insights into targeted interventions aimed at mitigating cell toxicity and restoring cellular function.

## 1. Introduction

Amyotrophic Lateral Sclerosis (ALS) and Frontotemporal Dementia (FTD) are two neurodegenerative diseases that present distinct clinical symptoms, but, in most cases, share some underlying features [1].

ALS is characterized by degeneration of upper and lower motor neurons, primarily in the brain and spinal cord, leading to progressive muscle weakness and atrophy [2]. Although the average disease onset age is between 65 and 75 years old, up to 10% of ALS cases involve younger patients with a disease onset below 45 years old [3]. In most cases, the earlier onset can be explained by the occurrence of mutations of target genes that can be transmitted genetically to the next generation. Thus, this 10% of ALS cases can be defined as familial ALS (fALS), while the remaining majority (90%) of ALS cases, which do not have any familial history, are therefore referred to as sporadic ALS (sALS) [4].

FTD is a neurodegenerative disease that affects the neurons of the frontal and temporal lobes, usually leading to impairments in executive functions, behavior, or language skills [5]. A fraction of the population affected by FTD (up to 33%) presents evidence of motor neuron degeneration and shares a similar genetic background as ALS patients [4,6], while 15% of ALS cases have an FTD co-diagnosis [7]. Both ALS and FTD share similar pathogenic mechanisms that lead to RNA and protein imbalances, as well as an altered cellular homeostasis, which strongly contribute to disease progression [8]. For these reasons, ALS and FTD are generally considered part of a disease continuum [7].

Like many other neurodegenerative diseases, such as Alzheimer’s disease (AD) and Parkinson’s disease (PD), the primary ALS-FTD feature is the presence of protein aggregates that are generally located in the cytoplasm and are associated with cellular toxicity [9,10,11]. Mammalian cells have evolved a sophisticated protein quality control (PQC) system that surveys the correct folding of proteins and detects proteins that are not correctly folded (unfolded, misfolded, and/or aggregated). In cooperation with the degradation systems, the ubiquitin–proteasome system (UPS), autophagy, and lysosomes, the PQC promotes the clearance of unwanted proteins, maintaining the so-called protein homeostasis or proteostasis. However, defects in this process, including the general decline of the proteostasis network during aging, can contribute to the accumulation of aggregated species [9]. In this review, we provide an overview of the main mechanisms that contribute to ALS-FTD.

### 1.1. Genes Associated with ALS-FTD

Even though fALS-FTD represents only a minor percentage of the total cases, mutations linked to the development of the disease can also occur in sALS-FTD cases. Extensive research throughout the past years led to the identification of more than 40 genes associated with ALS-FTD, and the list may keep growing [12,13,14,15,16].

The majority of those mutations were identified in genes involved in protein clearance pathways, RNA metabolism, cellular trafficking and protein homeostasis maintenance (Table 1) [6,17,18]. Despite the growing number and variety of these genes, four of them stand out as the most commonly mutated in ALS: *superoxide dismutase 1* (*SOD1*), *chromosome 9 open reading frame 72* (*C9orf72*), *fused in sarcoma/translocated in liposarcoma* (*FUS/TLS*), and *TAR DNA-binding protein 43* (*TARDBP-43)* [16,19,20,21].

In 1993, the analysis of 13 different fALS cases led to the identification of *SOD1* as the first gene to be involved in the development of the disease [22]. *SOD1* encodes for an important scavenger protein for oxidative species [23,24,25]. Mutations of this gene have important consequences on the physiological function of this protein: on one hand, they result in a gain of toxicity for SOD1, which becomes more aggregation-prone and tends to form pathological inclusions in the cytoplasm; on the other hand, they compromise its scavenger function, exposing the cell to a higher risk of damage caused by oxidative stress [26,27]. One of the consequences of oxidative stress is the formation of intra- or intermolecular disulfide bonds between the thiol groups of cysteine residues [28]; these covalent interactions may in turn promote the assembly and stabilization of molecular complexes that can lead to the formation of protein aggregates like those observed in the neurons of ALS-FTD patients [29,30,31,32].

Even though emerging as a later discovery, mutations of the *C9orf72* gene have quickly gained attention in the field, as they account for up to 50% of fALS-FTD cases [33,34]. These mutations are characterized by the repetition of a GGGGCC (G4C2) hexanucleotide sequence located in the first intron of the gene [35]. While most healthy individuals have ≤11 hexanucleotide repeats in the *C9orf72* gene, in pathological cases, the number of repetitions can range from 30 to several hundred [36]. Different mechanisms have been proposed to explain how the G4C2 repeat expansion may favor the development of ALS-FTD: (1) G4C2 repeats can assemble into secondary structures, either hairpin or G-quadruplex (G4) that would limit the accessibility of the transcript, therefore preventing its translation and reducing the expression of the physiological protein [37,38,39]; (2) the RNA transcripts containing the expanded repeat can cause toxicity through the formation of RNA foci that can sequester RNA-binding proteins; this in turn can interfere with the function of these RNA-binding proteins in RNA metabolism, while also promoting their co-aggregation with the structures formed by the G4C2 repeat expansion [37,40]; (3) these transcripts can still be used as templates for the repeat-associated non-ATG (RAN) translation, resulting in the production of dipeptide repeat (DPR) proteins, which are highly aggregation-prone species and contribute to cellular toxicity [41,42].

FUS is a predominantly nuclear protein that contains DNA- and RNA-binding domains and regulates the expression of several genes, such as *SOD1*, ubiquilin 1 and 2 (*UBQLN1* and *UBQLN2*) [43,44,45]. FUS also contains a low-complexity/prion-like domain, which is aggregation-prone [46,47]. Mutations in the *FUS* gene are responsible for around 5% of fALS-FTD and are usually located within the low-complexity/prion-like domain of the protein or its C-terminal nuclear localization signal (NLS), causing the accumulation and aggregation of the protein in the cytoplasm [48].

Similarly to FUS, TDP-43 is an RNA-binding protein involved in several aspects of RNA metabolism, including transcription, translation, splicing, and stability of mRNAs [49,50,51]. Also, TDP-43 contains an aggregation-prone disordered C-terminal region [52]. Most of the ALS-FTD-associated mutations have been identified in the disordered C-terminal region of TDP-43 and have been shown to aggravate its propensity to aggregate, especially in the cytoplasm [53,54,55]. This protein is of great interest in the study of neurodegenerative diseases, especially for ALS-FTD, because, independently of the familial or sporadic occurrence, it forms cytoplasmic neuronal inclusions in more than 95% of the cases; of note, TDP-43 aggregates have been documented even in the absence of specific genetic mutations, or in *C9orf72* ALS-FTD cases [56]. In the latter cases, it has been reported that glycine/arginine-rich (poly(GR)) proteins, one of the DPR species resulting from the RAN translation of the transcripts from *C9orf72* G4C2 repeat expansion, can sequester cytoplasmic TDP-43 and promote its aggregation; this finding was further supported by the observation that neurons of *C9orf72* ALS-FTD patients present pathological inclusions containing both poly(GR) and TDP-43 [57,58].

Considering that TDP-43 pathology is a key hallmark of ALS-FTD, this review aims to explore the current understanding of the mechanisms underlying TDP-43 dysfunctions and how they contribute to the disease.

### 1.2. Biological Processes Affected in ALS-FTD

A number of mechanisms have been involved in the pathogenesis of ALS. As discussed above, protein aggregation represents a common pathological feature of all ALS cases, supporting that defects in the protein quality control mechanisms, which are devoted to the maintenance of a healthy proteome, could contribute to ALS pathogenesis. This is further suggested by the finding of ALS-linked genes playing a role in protein degradation via either the proteasome (such as *UBQLN2*) or autophagy (*SQSTM1*, *OPTN*, *TBK1*) [13,59,60,61], as well as by the presence of poly-ubiquitinated proteins in the cytoplasmic inclusions that accumulate in ALS-FTD patient derived cells and cell models [62]. These aggregates also contain proteins that are not directly associated with the degradation pathways, including TDP-43, SOD1, and FUS, supporting the idea that the PQC system may be either overloaded or dysfunctional [63,64,65]. For example, DPRs deriving from RAN translation of mutant *C9orf72* transcripts can recruit and sequester proteasome components, leading to their inactivation [66]. Moreover, C9orf72 is also physiologically involved in autophagy, where it regulates Rab1a-mediated transport of the autophagy initiation complex to the phagophore [67]. Thus, the low expression levels of C9orf72 caused by its hexanucleotide repeat expansion could lead to a loss of function that ultimately impairs autophagy [67], indirectly promoting misfolded protein accumulation.

Additional mechanisms include defects in nucleocytoplasmic transport (NCT) in tissues from ALS patients and disease models [68,69,70]. Increasing evidence has shown that several elements of the nuclear transport machinery, including nucleoporins (Nups) and nuclear transport receptors (NTRs), are mislocalized in either nucleoplasmic or cytosolic aggregates in ALS [71,72,73]. Protein aggregates, including those enriched for TDP-43, FUS, or DPRs, have been shown to sequester components of the nuclear pore complex and the NCT machinery, leading to its dysfunction [68,70,74].

Next, defective axonal transport is a key pathological feature of ALS and has been implicated in disease onset and progression. Early neuropathological and electron microscopy studies of post-mortem ALS cases revealed abnormal accumulations of phosphorylated neurofilaments, mitochondria, and lysosomes in the proximal axon of large motor neurons [75,76,77,78]. The association between axonal transport disruption and ALS pathogenesis was further supported by studies in mutant SOD1 transgenic mouse models, which exhibited impaired kinesin-1 and dynein transport and reduced anterograde transport of cytoskeletal components before neurodegeneration onset [79,80,81,82]. This impairment was also observed in other ALS models, such as those associated with mutant TDP-43 and FUS [83,84,85]. For instance, both mutant TDP-43 and FUS have been reported to interfere with mRNA transport, leading to altered bidirectional movement and reduced delivery of key transcripts for local translation [86,87,88,89]. These findings collectively indicate that axonal transport impairments are among the earliest detectable pathological changes in ALS, emphasizing their potential role in disease initiation and progression.

Another common feature of ALS-FTD is mitochondrial dysfunction. Neurons and muscle cells are highly energy-demanding cells, and for this reason, they rely heavily on mitochondrial activity to sustain their activity and functionality [90]. ALS-mutations have been reported to increase TDP-43 mitochondrial localization, causing mitochondrial dysfunction [91]. Expression of wild-type or mutant TDP-43 and FUS fALS models has been shown to reduce the interaction between the vesicle-associated membrane protein-associated protein-B (VAPB) and protein tyrosine phosphatase-interacting protein-51 (PTPIP51) at ER-mitochondria tethering sites, leading to dysregulated Ca2+ signaling and impaired ATP production [92,93]. 

Additionally, studies in transgenic mice overexpressing human TDP-43 have revealed abnormal mitochondrial aggregation near the nucleus and disrupted mitochondrial dynamics, accompanied by alterations in key mitochondrial-associated membrane (MAM) proteins, such as mitochondrial fission protein 1 (Fis1) and mitofusin 1 (MFN1) [94]. Conversely, the downregulation of TDP-43 in HeLa cells reduces MAM density, suggesting that loss of TDP-43 function in ALS leads to MAM dysregulation and contributes to disease pathology [95]. Mutations in SOD1 impair mitochondrial Ca2+ homeostasis and lead to MAM dysfunction, further exacerbating mitochondrial deficits [96,97].

Another important consequence of mitochondrial dysfunction is the overproduction of reactive oxygen species (ROS), whose production is strongly enhanced by accelerated metabolism and cellular stress [98]. Oxidative stress seems to be a self-alimenting condition as it can trigger TDP-43 aggregation, which in turn can indirectly exacerbate mitochondrial stress and ROS production, via the sequestration of important regulators of the expression of nuclear genome (nDNA)-encoded mitochondrial constituents [99].

Glutamate excitotoxicity has for long been studied as a key mechanism in ALS pathogenesis, driven, in part, by impaired editing of the GluR2 AMPA receptor subunit [100]. In ALS patients, the efficiency of glutamine/arginine-rich (Q/R) site editing in the GluR2 subunit of AMPA receptors is significantly reduced in the ventral horn gray matter, leading to an increase in their calcium permeability [101]. Conversely, restoring arginine at the Q/R site of GluR2 prevents neurodegeneration, underscoring the importance of this modification in maintaining neuronal integrity [102]. Moreover, TDP-43 pathology correlates with reduced expression of the enzyme responsible for Q/R site editing, known as adenosine deaminase acting on RNA 2 (ADAR2), in motor neurons (MNs). This finding highlights a possible connection between TDP-43 aggregation and impaired GluR2 editing [103]. The cumulative effect of GluR2 hypo-editing and impaired calcium homeostasis contributes to motor neuron degeneration in ALS, highlighting potential therapeutic targets aimed at restoring ADAR2 function and reducing excitotoxic damage [100].

Multiple inflammatory pathways, both in the central nervous system (CNS) and periphery, have been shown to be dysregulated in ALS [104], as testified by the immune cells translocation into the CNS, altered cytokines production and abnormal peripheral immune cell population counts, including increased neutrophils and reduced regulatory T-cells (Tregs) [105]. The presence of ALS-FTD-related mutations, oxidative stress, and transcriptional deregulation is a factor that can contribute to the weakening of the DNA structure and potentially induce the activation of specific stress-responsive pathways, such as the cyclic GMP–AMP synthase (cGAS)–stimulator of interferon genes (STING) pathway [106,107,108,109]. Both TDP-43 pathology and C9ORF72 hexanucleotide repeat expansion have been shown to promote the activation of these inflammatory responses, providing a potential explanation for the phenotype observed [110,111]. Moreover, knockout of some inflammatory cytokines increased survival in a SOD1 mouse model [112], suggesting that inflammation is a potential therapeutic target [105].

## 2. TDP43 and Its Role in ALS-FTD

TDP-43 is encoded by the *TARDBP* gene, mapped to the 1p36.22 region of chromosome 1. TDP-43 is a ubiquitously expressed, evolutionarily conserved protein with affinity to both RNA and DNA. It is classified within the large heterogeneous nuclear ribonucleoprotein (hnRNP) family and, as such, it is able to bind RNA with considerable sequence-specificity [113,114,115].

**Figure 1:**
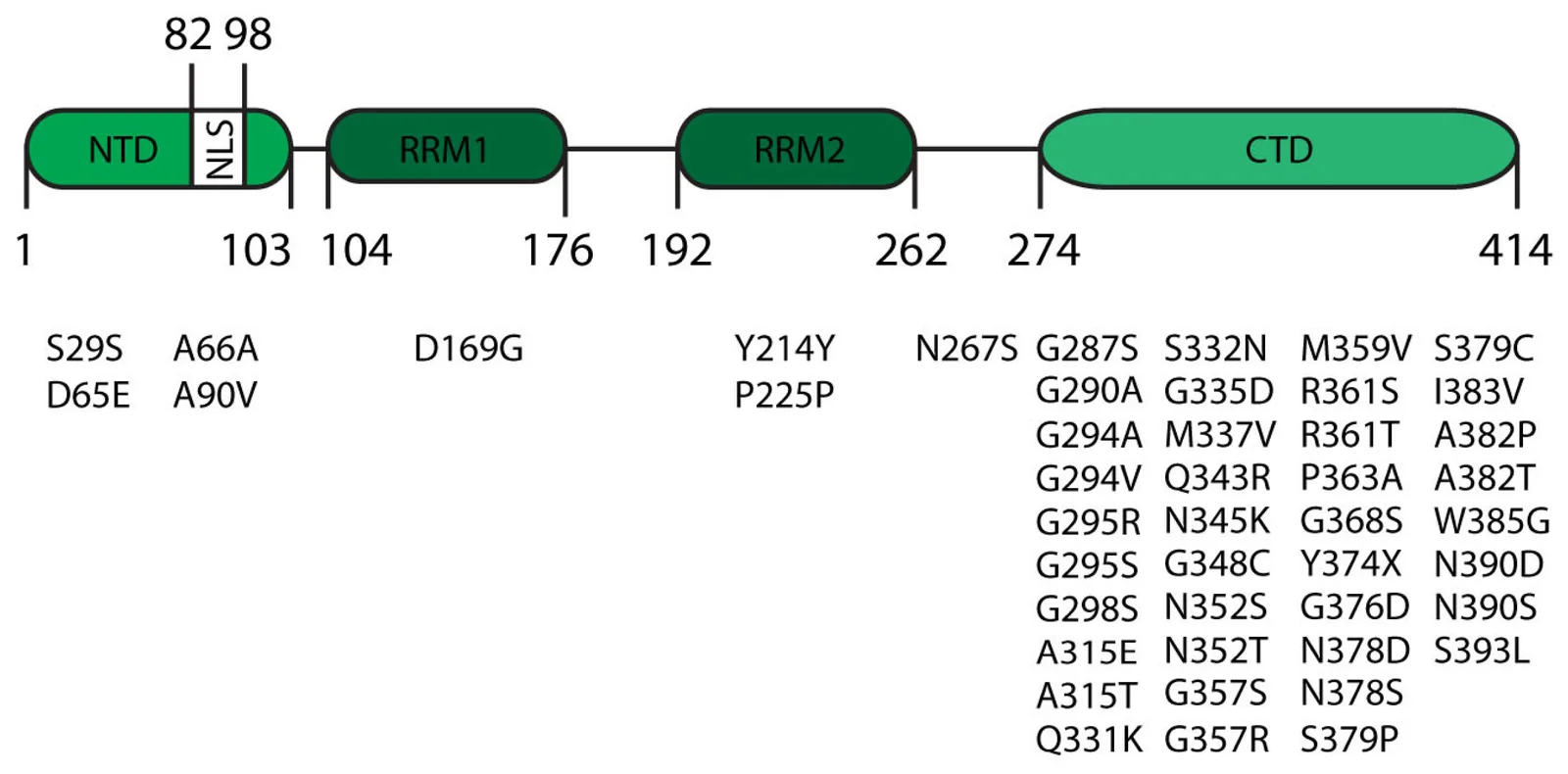
TDP-43 domain structure and the most known ALS-linked mutations [117,125,126,127,128,129].

Structurally, TDP-43 is composed of a well-folded N-terminal domain (NTD; residues 1–103), two highly conserved RNA recognition motifs (RRM1 and RRM2; residues 104–176 and residues 192–262, respectively), and a C-terminal domain (CTD; and residues 274–414), which encompasses a prion-like glutamine/asparagine-rich (Q/N) domain (residues 345–366) and a glycine-rich region (residue 366–414) (Figure 1) [116,117,118,119,120,121]. TDP-43 also possesses a putative nuclear export signal (NES, residue 239–250) localized inside the RRM2, although so far there is no evidence of an active nuclear export mechanism [122,123]. Due to its low solubility and high aggregation propensity, the full-length structure of TDP-43 has been challenging to characterize. Nevertheless, significant insights into its folding and structural organization have been gained through high-resolution analyses of its individual domains [116,117,119,120,124].

Under physiological conditions, TDP-43 predominantly exists as a dimer or dynamically transitions between monomeric and dimeric forms [130,131]. This property seems to be concentration-dependent, and it is linked to the ability of the NTD domain to drive oligomerization [132]. The NTD region contains a ubiquitin-like fold with one α-helix and six β-sheets, and it has been shown to promote the formation of functional homodimers, which are crucial for proper TDP-43 physiological function associated with nucleic acid binding [56,132]. On the other hand, single-molecule fluorescence techniques have shown that the NTD-mediated self-oligomerization may also favor the aggregation of TDP-43 via its intrinsically disordered C-terminal region [133]. Additionally, the NTD region contains an NLS (residues 82–98), which is recognized by Importin-α for active transport of TDP-43 into the nucleus [134]. Accordingly, mutations of this sequence in the NTD have been shown to enhance the translocation and aggregation of TDP-43 in the cytoplasm [135].

Within RRM1 and RRM2, there are two short, highly conserved sequence motifs that are essential for nucleic acid interaction, namely RNP-1 (KGFGFVRF in RRM1 and RAFAFVTF in RRM2) and RNP-2 (LIVLGL in RRM1 and VFVGRC in RRM2). The conserved RNP-1 and RNP-2 segments in TDP-43 are involved in binding to TAR DNA sequences and RNA sequences with UG-repeats [136,137]. Additionally, the RRM2 domain was shown to promote the dimerization of TDP-43 [138]. Regarding the nucleic acid-binding properties of these domains, their interaction with either single-stranded DNA (ssDNA) or single-stranded RNA (ssRNA) has been shown to increase TDP-43 solubility and, consequently, limit its aggregation propensity [139,140]. Importantly, through the RRM1 and RRM2 domains, TDP-43 actively recognizes and modulates the expression of a wide range of mRNA transcripts; the latter include the mRNA coding for TDP-43 itself, enabling an autoregulation mechanism to tightly regulate TDP-43 cellular concentration, possibly to maintain it within its solubility range [141].

The disordered CTD resembles the prion-like domains of numerous yeast proteins, such as Sup35, Rnq1, and Cyc8 [142,143,144]. The CTD of TDP-43 is aggregation-prone [145], and it harbors most of the ALS-associated mutations and phosphorylation sites. Finally, the TDP-43 C-terminal region can also undergo liquid–liquid phase separation (LLPS) to form dynamic protein droplets [146]. Mutations, persistent stress conditions, or aging contribute to the conversion of these droplets from a dynamic state into a solid-like state, facilitating the formation of irreversible pathological aggregates through a process that will be further discussed below [47].

TDP-43 prevalently resides in the nucleus, but it can also shuttle to the cytoplasm [55,147]. In the nucleus, TDP-43 is involved in transcription and splicing of messenger RNAs (mRNAs), as well as in maintaining RNA stability. In addition, TDP-43 regulates biogenesis of microRNAs (miRNAs) and processing of long non-coding RNAs (lncRNAs) [17,148]. TDP-43 shows high specificity towards UG-rich sequences of RNAs and, in the cytoplasm, can specifically target the 3′ untranslated regions (UTRs) of mRNAs/pre-mRNAs [17,49,56,149]. This suggests a broad role of TDP-43 in guaranteeing mRNA stability, maturation and transport [56,150,151,152], with important consequences spanning from the development of neuronal cells in the early stages of embryogenesis [115], to the maintenance of the neuromuscular junctions (NMJs), which progressively degenerate in ALS-FTD cases that show TDP-43 pathology [153,154,155]. Of note, TDP-43 can regulate the expression of the acetylcholinesterase (AChE), the glutamate receptor subunits, and the post-synaptic protein human disc large (DLG1), which are important elements for NMJ functionality [156,157]. TDP-43 is also implicated in the axonal transport of mRNA granules, contributing to the regulation of local translation. Aberrant TDP-43 axonal accumulation promotes the formation of mRNA granules that sequester important translation factors and nuclear-encoded mitochondrial mRNAs, thus depleting their local axonal expression [158,159,160]. Given that TDP-43 influences, directly and indirectly, fundamental cellular processes, it is not surprising that its dysfunction contributes to neurodegenerative diseases, including ALS, FTD and ALS-FTD, but also AD, PD, dementia with Lewy bodies (DLB), and limbic predominant age-related TDP-43 encephalopathy (LATE) [161,162,163,164].

### How TDP43 Properties Can Influence Its Behavior in ALS-FTD

As mentioned earlier, TDP-43 can form biomolecular condensates [165] via LLPS, a phenomenon by which a homogeneous liquid solution (or phase) of macromolecules separates into two different phases: one phase that is strongly enriched with a subset of molecules and another phase that is depleted of the same molecules [166]. The transient sequestration of molecules inside biomolecular condensates allows the generation of distinct microenvironments where specific biological activities can be regulated, such as transcription and signal transduction [167,168,169,170]. One example of this regulation mode is the activation of the signaling pathway through the receptor tyrosine kinase (RTK) fibroblast growth factor receptor 2 (FGFR2). This receptor was shown to phase separate together with a tandem Src homology 2 (SH2) domain-containing protein tyrosine phosphatase 2 (SHP2) and 1-phosphatidylinositol 4,5-bisphosphate phosphodiesterase gamma 1 (PLCγ1); this association, in turn, guarantees the efficient activation of the signaling cascade mediated through the activity of these enzymes [171]. Moreover, by concentrating and excluding different types of proteins and nucleic acids, these condensates can either enhance or inhibit biochemical processes, or they can buffer protein concentrations and regulate the recruitment of specific partners [172,173]. Importantly, LLPS is highly sensitive to changes in protein/nucleic acid stoichiometry that can occur in response to cellular signaling in physiological or pathological contexts [165,174,175].

Biological phase separation is driven by a summation of multiple weak interactions, e.g., electrostatic, hydrogen bonds, and cation–π interactions, and mainly relies on the multivalency of the molecules that take part in the process [174,176]. The valency of a protein relates to the number of interactions it can establish with other biomolecules [177]. Proteins involved in condensate biogenesis are commonly referred to as scaffold/driver, regulator, or client proteins. Scaffold proteins are the key drivers of condensate nucleation and are multivalent, while client proteins get recruited to phase-separate after nucleation and have lower valency [178]. Regulators typically trigger post-translational modifications (PTMs), modulating scaffold protein interactions with themselves or their clients, thereby influencing protein LLPS and condensate formation [179]. Similar to other RNA-binding proteins, TDP-43 undergoes LLPS in vitro [180,181], and evidence exists that certain functions of TDP-43 are closely related to its ability to phase-separate. For instance, a recent study demonstrated that the association of TDP-43 with specific RNA regions across the transcriptome depends on its condensation behavior, which consequently affects its RNA processing functions [146]. Further highlighting the critical role of TDP-43 phase separation, especially in the brain, a mouse model expressing a TDP-43 variant unable to undergo LLPS exhibits defects in neuronal activity and translation regulation [182].

Structurally, TDP-43 contains specific features that allow establishing a network of interactions that involve the intrinsic disordered regions (IDRs) of proteins and their RNA-binding domains. IDRs are a hallmark of phase-separating proteins lacking secondary or higher-order structures imparted by large stretches of low sequence complexity, which generally include high concentrations of polar and aromatic residues, including arginines and tyrosines [183]. IDRs establish transient intermolecular interactions that allow for the dynamic rearrangement of the internal structural components, promote the free fusion of smaller droplets into a larger droplet, and ensure the reversible remodeling upon removal of the external shear forces [47].

In addition to proteins, RNA is another macromolecule that plays a key structural role in condensates, especially in RNP granules. Recent evidence suggests that RNA is an ideal scaffold molecule because it is long, flexible, and multivalent and it can interact through specific intermolecular base-pairing events: RNA–RNA interactions are at the basis of condensate self-assembly and the strength of intermolecular RNA–RNA interactions will define the properties of each RNA condensate [184]. It is well known that RNA-binding domains, including RRMs, promote RBP multivalency and thus RNA-binding can regulate the LLPS dynamics of its protein interactors [185,186,187,188]. Concerning TDP-43, it was shown that RNA influences in a sequence- and length-specific manner TDP-43 LLPS: UG-rich RNAs induced the formation of TDP-43 droplets in vitro as the length of the oligonucleotide increases [189].

A specific subset of condensates where RNA plays a crucial role are ribonucleoprotein (RNP) granules, which are implicated in the regulation of several aspects of the RNA life cycle. Each RNP granule has a unique composition in terms of RNA and RNA-binding proteins and forms in response to different stimuli [190]. Examples of RNP granules comprise nucleoli, Cajal bodies in the nucleus, and stress granules (SGs) and P-bodies in the cytoplasm, but also protein-containing condensates such as PML nuclear bodies [165]. SGs, which can recruit TDP-43, are considered as “transient storage and sorting stations” for RNA binding proteins, translationally stalled mRNAs, and arrested pre-initiation complexes [191].

Recent studies were aimed at identifying the features of TDP-43 implicated in LLPS. In general, amino acid motifs with low sequence complexity can facilitate LLPS: examples include the tyrosines flanked with glycine or serine in the FUS protein [192], the large number of phenylalanine/glicine (FG) and arginine/glycine (RG) dipeptide repeats in the DEAD-box helicase 4 (Ddx4) [193], and the arginine-rich dipeptide repeats in C9orf72 [194]. In these proteins, the formation of multivalent connections is facilitated by the presence of dozens of these motifs. In contrast, there is no dominant LLPS motif in the low-complexity domain (LCD) of TDP-43 CTD, despite its ability to form condensates. TDP-43’s LCD is composed of 160 residues, and it contains only six positively and three negatively charged amino acids, four typical (glycine/serine)-(phenylalanine/tyrosine)-(glycine/serine) LLPS motifs, and three sparsely distributed FG repeats [195,196]. Li et al. reported that TDP-43 LLPS is driven by just three tryptophan residues, with minor contributions from one tyrosine and three phenylalanines [181]. They also suggested that only a few residues may be required for TDP-43 LLPS because of the α-helical segment (spanning ∼20 residues) in the middle part of the CTD that tends to self-assemble, reducing the number of motifs required for creating a multivalent connection [181]. Deleting this α-helical region or reducing its secondary structure propensity by point mutations or by inserting random sequences has been shown to abrogate LLPS of TDP-43 [53,197].

To date, more than 50 ALS-FTD-associated mutations have been identified in the *TARDBP* gene (Figure 1) [125,126,127,128,129,198]. Some of them are localized inside or nearby the NLS region and promote the cytoplasmic re-localization, and subsequent aggregation, of TDP-43 [134,135]. For instance, the ALS-associated TDP-43 variant harboring the A90V mutation within the NLS tends to form insoluble cytoplasmic aggregates, which can also incorporate and sequester the endogenous TDP-43 protein [134]. A few other mutations have been identified in the RRMs, impairing their RNA-binding activity, but most of their disease-linked mutations reside in the disordered CTD [56]. Another sensitive region for ALS-related mutations is the TDP-43 C-terminal region [52]. As demonstrated both in vitro and in cellular models, mutations within this domain, such as G294A, Q331K, M337V, Q343R, N345K, R361S, N390S, and N390D, strongly increased TDP-43 aggregation and cytotoxicity (Figure 1) [52,198]. Moreover, the mutant TDP-43 A315T has been reported to form amyloid fibrils in vitro and provoke neuronal death when added to the cultured cells [199]. In addition, various peptides bearing pathogenic TDP-43 mutations, such as G294V, G294A, and G295S, have been found to form twisted amyloid-like fibrils [200]. Together, these findings highlight the importance of the CTD of TDP-43 in promoting its aggregation.

## 3. Origin of Protein Aggregation

As mentioned earlier, several neurodegenerative diseases, including ALS and FTD, are characterized by pathological deposition of aberrant protein aggregates in cerebral tissues and/or in the spinal cord [201]. The aggregation of a protein can contribute to toxicity with two, not mutually exclusive, mechanisms: loss of function, which occurs through the impairment or the complete abrogation of the physiological activity of the misfolded protein, and gain of function, which is promoted by metastable proteins that undergo aggregation in a process associated with cytotoxicity [9,202].

Protein aggregation is a highly complex process, and the mechanisms that lead to the formation of insoluble amyloid aggregates are not fully understood.

Emerging evidence has reported that proteins exhibit a complex phase behavior, as they can populate the native, droplet, and amyloid states [172,174]. As a consequence, multiple pathways can contribute to the conversion between the native and amyloid states of proteins [203]. A direct deposition pathway and a condensation pathway have been described as two alternative models for the protein aggregation mechanism [202].

Along the direct deposition pathway, proteins convert from the native state to the insoluble amyloid state through a multistep mechanism that includes the formation of misfolded proteins and their assembly into increasingly ordered oligomeric species, and can culminate with the transition into amyloid aggregates [204,205,206]. Along the condensation pathway, the impairment in the phase behavior of proteins can promote the transition from a liquid-like droplet state into an intermediate gel state, which can mature into a more stable type of amyloid-like aggregates. The stability of these different states, as well as the conversion rates between them, can vary depending on the protein [165,203].

Currently, it is not well established why some proteins proceed down an aggregation pathway through a liquid intermediate and why others directly aggregate from solution. It is also conceivable that the same protein can follow either pathway depending on its concentration or the environmental conditions in the cell. Presumably, changes in physical or chemical conditions or genetic mutations can also push proteins down these different pathways, although this remains to be shown in most cases [165,203].

To ensure a correct proteome, cells have evolved a sophisticated PQC system, in which molecular chaperones guide the nascent polypeptide chain during translation on the ribosome and subsequent folding to prevent or reverse misfolding and aggregation [206]. They also cooperate with the protein degradation systems in an interconnected network to guarantee a balanced proteostasis [204,205,206]. Molecular chaperones can act at different steps of the aggregation process, by either preventing the initial assembly between non-native monomeric proteins into progressively larger oligomers, or even by promoting the disaggregation of amyloids, once formed [206]. Hsp70 and Hsp90, for example, can recognize and actively refold misfolded proteins back to their native conformation [207,208]. Alternatively, molecular chaperones, especially small HSPs (sHSPs), can act as “holdases”, temporarily sequestering misfolded proteins to limit their toxicity and facilitate their subsequent handling by ATP-dependent chaperones of degradation systems [9,209]. Other molecular chaperones, such as the Hsp110/Hsp70/Hsp40 complex, are able to disentangle aggregates in an advanced state, favoring their solubilization and clearance [210,211,212]. A failure of any of these systems, caused either by genetic mutations or an overwhelming production of toxic species, can inevitably tip the balance towards the accumulation of misfolded and aggregation-prone proteins [9,213]. In turn, whether via the direct deposition pathway or through the condensate-mediated pathway, these events can lead to the deposition of the protein aggregates observed in the neurons of ALS-FTD patients.

### 3.1. Deposition Pathway in Protein Aggregation Mechanism

The majority of proteins must be folded into defined three-dimensional structures to acquire functional activity. However, protein chains can adopt a multitude of conformational states, and their biologically active conformation - the native state - is often only marginally stable under physiological conditions [9,214,215]. Mechanistically, protein aggregation is driven by non-native interactions that occur between sensitive regions in the protein primary sequence that are usually enriched for hydrophobic or charged amino acids [215,216,217,218]. Proteins can undergo inherent conformational fluctuations [219] or can experience local structural perturbations [220,221] that allow partial exposure of these sensitive regions – or “hot-spots” – that are usually buried under physiological conditions as they tend to establish promiscuous interactions [216,217,222].

The majority of proteins, if not all, in the cellular context require assistance during the folding process to reach their functional structure at a biologically relevant rate. The folding process itself is prone to error, giving rise to misfolded states and off-pathway aggregates that are linked with neurodegeneration in several disorders [9]. Usually, the native protein conformations are thermodynamically stable, and the linear amino acid sequence encodes all the required information to fold properly [222]. However, due to the challenging environment, including the macromolecular crowding, the concentration of each given protein, which can be close to its solubility threshold [223], and subtle changes in the environmental conditions, proteins may adopt partially folded or misfolded conformations [224]. These partially folded or misfolded conformations have a great tendency to collapse and form toxic aggregates that may be more thermodynamically stable than the native structure [204,225].

Concerning TDP-43, several studies have highlighted how these different factors can influence its aggregation propensity. Experimental evidence indicates that the NTD of TDP-43 is crucial for the acquisition of its native conformation: computational modeling suggests that the deletion of just the first 10 amino acids of TDP-43 can cause the misfolding of this domain [130]. Moreover, the folding state of the distinct TDP-43 domains is sensitive to environmental changes, facilitating the formation of non-native interactions that drive initial oligomeric assembly, which may subsequently progress into aggregation [226,227,228].

### 3.2. Condensate-Mediated Pathway in Protein Aggregation Mechanism

As mentioned earlier, aberrant phase transitions have been reported for many of the proteins associated with ALS-FTD, including TDP-43, FUS, hnRNPA1, and hnRNPA2/B1, and are thought to contribute to cytotoxicity [47,53,180,229,230,231,232].

Hence, dysregulation in the formation, maintenance, or clearance of these assemblies may provide a stepping-stone for pathological aggregation [233,234]. Indeed, spontaneous maturation of dynamic protein droplets and hydrogels to solid aggregates has been observed over the course of hours in the test tube and in cells [47,180,233,235,236]. These phase transitions are intrinsically related to the metastability of the condensates, meaning that their soluble state can be maintained only within certain conditions and for a determined period of time before phase transition [233,237].

The “aging” process of biomolecular condensates is a phenomenon by which their physical properties move from a liquid to a less dynamic state that can be reverted as long as the new intra- and intermolecular interactions developed are not too stable [186,238]. For example, it has been demonstrated that increasing the concentration of FUS in solution promotes its phase separation from the solvent and, consequently, drives the formation of liquid-like droplets in vitro; however, once the protein concentration in the liquid compartments reaches a critical threshold, it can trigger aggregation and lead the transition from liquid to a solid-like state [47]. Moreover, recent studies reported that ALS-FTD-linked mutations in TDP-43 or other RBPs containing IDRs enhance the rate of the condensate-aging process [236]. Many of these mutations map inside the IDRs of these RBPs [47,180,236] and are thought to change their conformational landscape, presumably by promoting amyloid-like interactions that ultimately lead to protein aggregation [46,180,239,240]. This suggests that even the wild-type versions of prion-like RBPs have a high propensity to form aberrant condensates, likely because of protein misfolding and aggregation [165].The fact that these liquid-to-solid transitions are enhanced by disease mutations further highlights the relevance of phase transitions to pathology [47,241]. 

Besides disease-causing mutations, aberrant PTMs on RNA-binding proteins are also related to pathological phase transition in several neurogenerative conditions, including ALS and FTD; the most prominent example is represented by the accumulation of hyperphosphorylated TDP-43 in the patients’ brain cells [242,243]. The role of PTMs has become widely studied in the past years, as they can alter protein valency and interaction strength to either promote or inhibit phase separation [244]. Understanding how a specific PTM influences the aggregation of TDP-43 (and other ALS-FTD-linked proteins) is fundamental for the design of therapeutic approaches. Thus, several PTMs have been studied so far. One example is represented by TDP-43 phosphorylation, even though defining the ultimate impact of this PTM is challenging: hyperphosphorylation of its serine residues in the LCD is a common feature of pathological inclusions in patients, and experimental conditions that promote TDP-43 phosphorylation have been shown to promote neurotoxicity [245,246,247,248]. However, recent data demonstrated that C-terminal TDP-43 phosphorylation suppresses its phase separation and aggregation both in vitro and in cells, likely by altering the electrostatic interactions that contribute to this process [249].

The liquid properties of condensates can also be maintained thanks to the action of molecular chaperones. Chaperones can modulate the internal structure, dynamics, and other in situ features of condensates [250,251,252]. Chaperones seem to “fluidize” the structure by increasing the rate at which the condensate can internally reorganize. In various conditions, this fluidization seems to be crucial for maintaining condensates in a material state that is rapidly dispersible [209,253,254,255,256,257]. In addition, recent experimental evidence suggests that misfolding-prone proteins, including TDP-43, accumulate in condensates and promote condensate hardening [194,250,258]. Firstly, it has been proposed that condensates may change the kinetics of protein aggregation by inducing the formation of a rate-limiting nucleus that serves as a sink for protein misfolding and aggregation [259]. Secondly, misfolded proteins could specifically bind to the proteins contained in condensates and establish long-lasting physical interactions that alter condensate dynamics [172]. Third, misfolded proteins, including defective ribosomal proteins (DRiPs) can accumulate inside SGs and other types of biomolecular condensates, including PML nuclear bodies and nucleoli, converting them into an aggregated state; this process is prevented by the action of the HSP70 and HSP90 chaperones, as well as by the valosin-containing protein (VCP), whose mutations are associated with ALS-FTD [250,260,261,262].

## 4. How Condensates Can Contribute to Protein Aggregation: Stress Granule Formation and ALS Protein Aggregation

Conditions that push the PQC system to reach a saturation limit, as it can happen in aging cells or in the neurons of ALS-FTD patients, may result in the accumulation of toxic species that cannot be readily disposed of [263]. In this case, the cell can adopt other strategies to keep these potential threats at bay when the system is overloaded. One of these mechanisms is the sequestration of aggregation-prone proteins into specific compartments, such as aggresomes, juxtanuclear quality control compartments (JUNQs), insoluble protein deposits (IPODs), and Q-bodies [264,265]. The compartmentalization of unfolded/misfolded species represents a temporary strategy that might facilitate the subsequent engulfment of aggregated proteins by the autophagic pathway [266]. The existence of these physiological compartments poses the question of whether the inclusion bodies that are present in the neurons of ALS-FTD patients could represent a first attempt by the cell to spatially confine misfolded and aggregated proteins to protect the rest of the cell from their toxicity.

Apart from these specific structures, other subcellular compartments whose primary function is not the containment of misfolded proteins can still become sites for their accumulation. Of particular interest in the field of ALS-FTD and other neurodegenerative diseases in this sense are RNP granules [18]. Their connection to ALS-FTD derives from the fact that these condensates are enriched for RBPs, including TDP-43, hnRNPA1, FUS, and T-cell intracellular antigen 1 (TIA), whose aggregation propensity could promote the irreversible maturation of these granules [202]. For these reasons, the role that RNP granules play in the formation of protein aggregates has long been debated. It is the case, for example, of SGs [234,267]. SGs are membraneless compartments that readily assemble in the cytoplasm upon stress and serve protective purposes for the cells, including RNA safeguarding and indirect regulation of intracellular signaling pathways through sequestration of specific molecular players [268,269,270]. SG assembly occurs through a process of LLPS that is triggered by multivalent interactions between the Ras GTPase-activating protein-binding protein (G3BP) and free mRNA that is released from disassembling polyribosomes [271]. Mature SGs contain a concentrated core, particularly enriched for G3BP1 and RNAs, surrounded by a more dynamic and compositionally varied shell. The components of this outer layer can rapidly exchange with the surrounding cytoplasm, often facilitated by energy-dependent molecular chaperones [272]. This continuous exchange, combined with the constant supervision of molecular chaperones, guarantees the dynamic properties of SGs [250,260]. Different proteomic studies have tried to identify the proteins that can be recruited inside SGs and understand how they can regulate their assembly and dynamics [272,273,274,275]. Many of these proteins are involved in RNA metabolism, translation regulation, post-translational modifications, PQC system [250,272,276]. Some of these components are required for the maintenance of condensate dynamics [190]. For example, DRiPs that are released in large amounts upon polyribosome disassembly [277] can accumulate inside SGs, converting them into a less dynamic state. Several chaperone complexes, including the HSPB8-BAG3-HSP70 complex and VCP in cooperation with Zinc Finger AN1-Type Containing 1 (ZFAND1), a protein containing a ubiquitin-like (UBL) domain, prevent the accumulation of DRiPs and other misfolded proteins inside SGs, promoting their clearance and ensuring SG disassembly [260,278,279]. Defects in the degradation pathways, as demonstrated by the depletion of VCP or BAG3-HSPB8, can cause the accumulation of DRiPs in the proximity of SGs and affect their dynamics [280].

Besides DRiPs, many other aggregation-prone proteins, including TDP-43, can accumulate within SGs upon stress and can become targets for degradation via ubiquitination to minimize the risk of aggregation and facilitate the recovery after stress [251,281]. On one hand, the recruitment of these proteins to SGs and other biomolecular condensates can be beneficial for their containment during stress, at least in early stages [264]. This behavior can partially be explained by the ability of SGs to concentrate important elements for the maintenance of proteostasis, such as molecular chaperones and enzymes that mediate post-translational modifications of proteins. Increasing experimental evidence, indeed, shows that molecular condensates can enhance reaction rates by concentrating enzymes and their substrates within the same space, thereby facilitating the interaction with their molecular targets [282,283]. Moreover, several studies have shown that TDP-43 aggregates are usually excluded from SGs and that the fraction of TDP-43 that is contained inside SGs is dynamic, supporting the idea that SGs may protect TDP-43 from irreversible aggregation [284,285,286]. On the other hand, the progressive accumulation of misfolded and aggregation-prone proteins inside these condensates, including the accumulation of DRiPs, paired with defects of the PQC system, can promote the conversion of the dynamic liquid-like SGs into a solid-like state [202,234,287]. This property has led to the hypothesis that a similar mechanism can contribute to the formation of ALS-FTD pathological inclusions [288].

These two apparently contrasting observations do not necessarily contradict each other, but rather, they highlight the complexity of interactions that need to be finely tuned to maintain SG and protein homeostasis and that, if disturbed, would contribute to the downhill aggregation effect observed in the post-mortem neurons of ALS-FTD patients.

## 5. Post-Translational Modifications as Potential Modifiers of Protein Aggregation

PTMs can modulate protein function, fate, and turnover, as well as aggregation propensity and the LLPS behavior [179,289,290]. Moreover, PTMs have also been shown to finely regulate the activation of stress-responsive pathways, like in the case of ubiquitination for the autophagy-mediated degradation of aggregated proteins and damaged SGs [291,292,293,294]. The deregulation of these systems has a strong impact on the solubility of proteins and can explain why the inclusion bodies found in the patients’ cells often contain heavily modified proteins, such as, for example, hyperphosphorylated and/or ubiquitinated TDP-43 [247,295]. Being the main aggregated species found in ALS-FTD protein inclusions, in this section, we focus on the effects of PTMs on TDP-43 behavior and their potential role in enhancing or protecting against aggregation.

**Figure 2:**
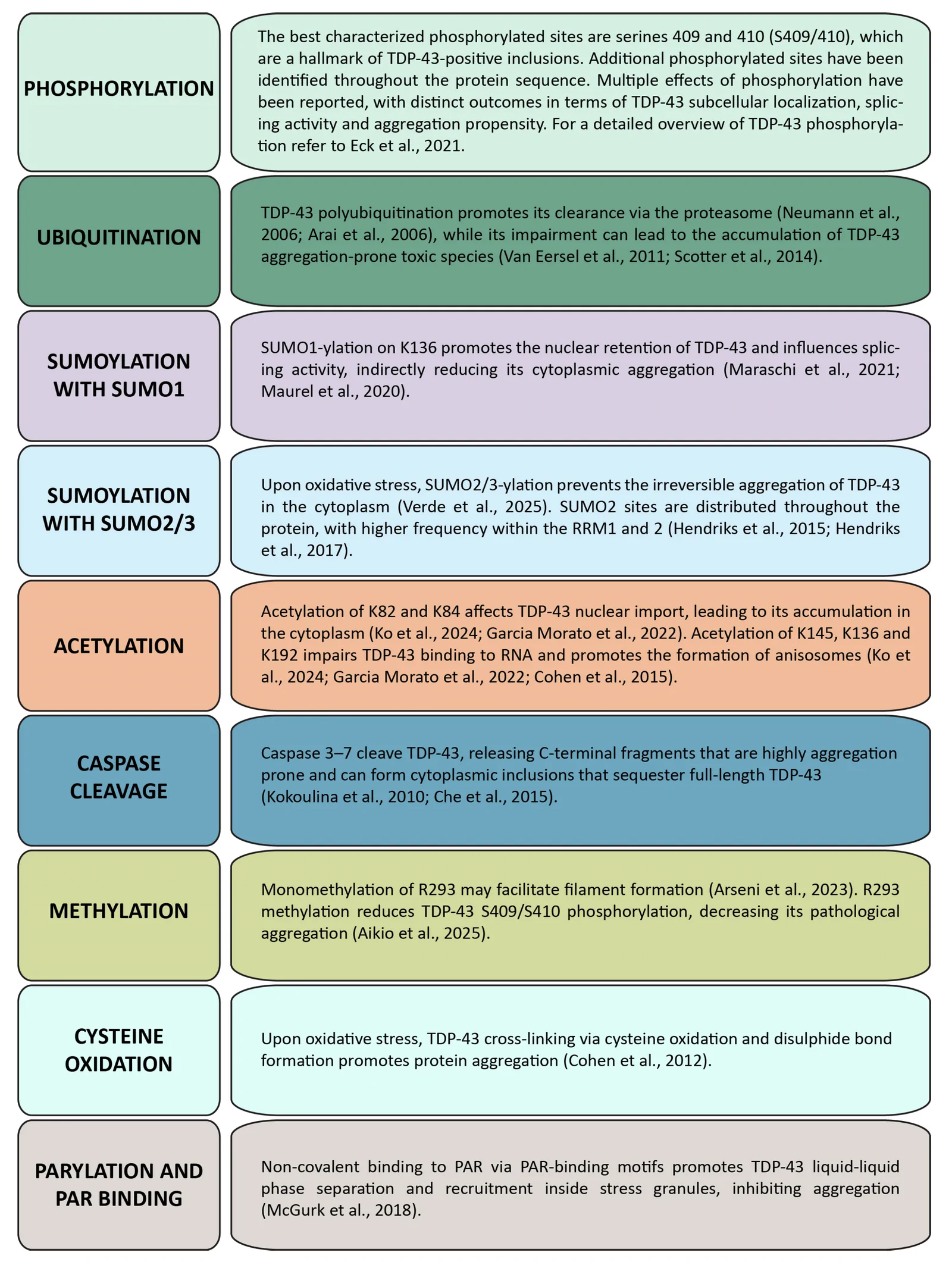
Known post-translational modifications of TDP-43 and their impact on protein function and aggregation-propensity [285,286,295,296,297,298,299,300,301,302,303,304,305,306,307,308].

TDP-43 can be ubiquitinated, phosphorylated, acetylated, methylated, modified with poly (ADP ribose) (PAR), oxidized, fragmented by caspase, and also conjugated to SUMO1 and SUMO2/3 (Figure 2) [122,241,281,285,286,295,296,297,298,299,300,301,302,303,304,305,306,307,308].

### 5.1. TDP-43 and Phosphorylation

Hyperphosphorylated TDP-43 aggregates are considered a hallmark of ALS-FTD, in particular, the phosphorylated residues Ser-379, Ser-403, Ser-404, and Ser-409/Ser-410 are the most recurrent on ALS-FTD TDP-43 inclusions [247,308]. Additionally, several ALS-FTD-linked mutations of TDP-43 result in amino acid substitutions that introduce or delete phosphorylation sites, and influence TDP-43 behavior in terms of localization, splicing activity, and aggregation propensity [241]. For a long time, phosphorylation has been considered an enhancer of protein aggregation [56,245,248,309]. Nonetheless, most recent research has shown that phosphorylation of specific residues may, on the contrary, impair TDP-43 LLPS and aggregation [249,310,311,312]. The introduction of phosphate groups can alter the charge of the residues, resulting in the formation of repulsive electrostatic forces between negative charges [313]. It was demonstrated that phosphomimetic mutations of several residues that are in close proximity in the C-terminal domain effectively reduced TDP-43 phase separation and aggregation [249,311]. The intermolecular interactions between the CTDs of different TDP-43 molecules are thought to be the main factor responsible for its aggregation [53,54]; thus, the amount of phosphorylation within these critical residues may limit the development of these aberrant interactions. On the other hand, the modification of residues that are localized in other TDP-43 domains can promote its aggregation through a different mechanism and would explain the different conclusions derived from different studies.

### 5.2. TDP-43 and Ubiquitination

The accumulation of poly-ubiquitinated insoluble TDP-43 is also a very common feature of pathological inclusion in ALS-FTD, likely caused by an impairment of the protein degradation systems [295,305,306,307]. Beyond increasing TDP-43 insolubility, polyubiquitination has also been shown to promote its cytoplasmic mislocalization and nuclear depletion [314,315,316]. TDP-43 contains numerous lysine residues that can be targeted by ubiquitination, although mass spectrometry studies and site-directed mutagenesis approaches have revealed that all of them can contribute equally to the modification of the protein [317]. However, lysine-to-arginine (K-R) substitutions have uncovered a possible relationship between ubiquitination and other PTMs. Notably, one study demonstrated that the K408R mutation enhances phosphorylation at Ser-409/410 within a C-terminal TDP-43 fragment [317]. These findings underscore how dysregulation of TDP-43 ubiquitination may influence multiple cellular pathways, ultimately contributing to disease pathology.

### 5.3. TDP-43 and SUMOylation

Similarly to ubiquitination, SUMOylation has gathered new attention in the past years as a modifier of protein solubility. Previous work showed that TDP-43 inclusions can also contain SUMO2/3 [318]; nevertheless, our understanding of whether/how SUMOylation regulates TDP-43 function, turnover, and aggregation behavior is still in its infancy. Prediction algorithms identify a few SUMO residues in TDP-43, namely K84, K95, K97, K121, K136, K160, K176, K192, K263 and K408 that can be potentially modified by either SUMO1 or SUMO2 [319,320,321,322]. In parallel, advanced mass proteomic studies identified up to 16 SUMO2 sites in the TDP-43 protein [303,304,323,324]. Modification of a target protein with SUMO1 or SUMO2/3 is generally not interchangeable and is associated with different functional outcomes. SUMO1-ylation involves the conjugation of a single moiety of SUMO1 to a specific set of targets, usually with a regulatory function, as in the case of Ran GTPase-activating protein (RanGAP1). The attachment of SUMO1 to RanGAP1 promotes its association with the nucleoporin/SUMO E3 ligase RanBP2 at the nuclear pore complex, where it facilitates the nucleocytoplasmic trafficking by promoting the Ran GTPase cycle [325,326]. SUMO1-ylation has also been implicated in the formation of nuclear condensates known as Promyelocytic Leukemia Nuclear Bodies (PML-NBs). The PML protein contains both a SUMO consensus sequence and a SUMO-interacting motif (SIM); when PML proteins get modified with SUMO1, the formation of SUMO-SIM interaction promotes their phase separation into PML-NBs [327,328].

Contrary to SUMO1, SUMO2/3 molecules are more often found in a free state under normal conditions, but they are readily conjugated, usually as K11-poly-SUMO2/3 chains, to a various set of protein substrates upon stress [329,330,331]. This modification has been suggested to increase the solubility of a wide range of substrates, especially in the early stages of the stress response, likely in an attempt to protect unfolded proteins that are at particular risk for aggregation if they are not immediately targeted for degradation [332]. Yet direct evidence of the impact of SUMO2/3-ylation on the “solubility” of single proteins in cells is still lacking.

The role of SUMOylation on TDP-43 has been only marginally studied so far. A recent study showed that TDP-43 can be conjugated with SUMO1 at lysine residue 136; this modification promoted the nuclear retention of TDP-43, indirectly reducing its cytoplasmic aggregation [296,297]. Recent attempts were made to investigate the role of TDP-43 SUMOylation in a mouse model. Mutating the K residue at position 408, one of the putative SUMOylation sites, into an arginine (R) caused significant age-dependent changes in motor, social, and cognitive functions that resembled the symptoms associated with ALS-FTD. The authors suggested that TDP-43 SUMOylation at K408 with SUMO2 may play a protective role [333]. We recently published that TDP-43 becomes a target for SUMO2/3-ylation under oxidative stress conditions [286]. Importantly, stress-induced SUMO2/3-ylation of TDP-43 prevents its irreversible aggregation in the cytoplasm [286]. We also identified protein inhibitor of activated STAT protein 4 (PIAS4) as the specific SUMO E3 ligase responsible for this modification. The knockdown of PIAS4 promoted TDP-43 aggregation in the cytoplasm upon stress, similarly to what was observed upon general SUMOylation inhibition [286]. PIAS4-mediated SUMO2/3-ylation of TDP-43 is activated upon stress, when a pool of TDP-43 molecules, especially those that translocate into the cytoplasm, is unbound to RNA [286]. RNA binding is indeed an important regulator of TDP-43 stability, as it limits the intermolecular interactions that drive its phase separation and possibly aggregation [136,284,334,335]. Intriguingly, although the SUMO2 sites are distributed throughout the protein sequence, the vast majority is localized within the two RRMs [303,304]. Moreover, the efficiency of TDP-43 conjugation with SUMO2/3 by PIAS4 decreases in the presence of higher concentrations of UG-rich RNA, which binds with high affinity to TDP-43, demonstrating that RNA and SUMO2/3 compete for the same residues [286]. Finally, upon RNA loss, cells induce TDP-43 SUMO2/3-ylation to prevent its irreversible aggregation [286]. All together, these findings clearly suggest that conjugation of SUMO2/3 chains to TDP-43 is a protective mechanism that cells put in place to prevent its irreversible aggregation. This may have important implications for disease progression. In fact, we found that the truncated and aggregation-prone TDP-35 and TDP-25 fragments, which accumulate in the inclusion bodies in the patients affected by ASL-FTD, are poorly SUMO2/3-ylated [286]. Moreover, analysis of post-mortem α-motor neurons (MNs) of ALS-FTD patients showed an inverse correlation between PIAS4 immunoreactivity and the presence of TDP-43 pathological aggregates, further suggesting that a dysregulation of PIAS4-mediated SUMO2/3-ylation of TDP-43 may contribute to protein aggregation during the course of disease [286]. Future studies are required to understand to what extent defects at the level of SUMOylation and/or PIAS4 expression and subcellular localization may contribute to ALS-FTD pathogenesis and progression and to clarify whether similar mechanisms may apply to other RBPs that aggregate in ALS-FTD, such as FUS and hnRNPs [17,46,47].

### 5.4. TDP-43 and Acetylation

TDP-43 lysine residues can also be targeted for acetylation, with yet unclear consequences for this modification. Acetylation of K82 and K84, located near the NLS of TDP-43, has been shown to affect its nuclear import, leading to the accumulation of TDP-43 in the cytoplasm [336,337]. Previous studies have demonstrated that, in response to stress, TDP-43 can also be acetylated on K145, K136, and K192 and that this modification both impairs RNA binding and promotes the self-assembly of TDP-43 into nuclear condensates, known as anisosomes, whose liquid-like material properties are guaranteed by the activity of HSP70 chaperones [337,338,339]. Acetylated TDP-43 has also been found in the inclusions of ALS patient spinal cord, opening the question of whether this PTM can be somehow connected to the formation of TDP-43 aggregates in the disease [338].

### 5.5. TDP-43 and Methylation

Other types of PTMs can potentially take place on TDP-43 but are currently poorly studied, such as arginine methylation. Mass spectrometry studies have reported three methylated residues: R42, R275, and R293 [300,340,341,342]. Nonetheless, the functions and regulatory mechanisms of these modifications remain greatly unexplored. A very recent study reported that the protein arginine methyltransferase 1 (PRMT1) mediates the methylation of TDP-43 at R293 and that this modification reduces p38α MAPK-driven phosphorylation at S292 and S409/S410, which in turn attenuates TDP-43 aggregation in human neuronal SH-SY5Y cells [299]. Future efforts should aim at a better understanding of the impact of different PTMs on TDP-43 aggregation propensity and functionality, as well as on the cross-talk between different PTMs that are often simultaneously regulated in the cells that constantly face and respond to a variety of challenges and stimuli.

### 5.6. TDP-43 C-Terminal Cleavage

Together with wild-type misfolded TDP-43, ALS-FTD inclusions are enriched for fragmented TDP-43 that derives from caspase cleavage at several sites of the full-length protein [295,301]. While the above-mentioned PTMs are in principle reversible by the action of specific enzymes, caspase-mediated cleavage of TDP-43 is a modification that can’t be reverted [343,344]. This cleavage produces N-terminal fragments of TDP-43 that are degraded quickly, leaving two possible C-terminal containing species, generally referred to as TDP-43 C-terminal fragments (CTFs): a 35 kDa fragment (TDP-35) lacking only the NTD and the NLS but is still correctly folded and can bind RNA; a 25 kDa fragment (TDP-25), which lacks the NTD, NLS and most of RRM1 [302,344]. The absence of a functional NLS shifts the localization of these two truncation variants, which tend to accumulate and aggregate in the cytoplasm [134,302,345].

## 6. Conclusions

In the previous sections, we have described how different pathways and mechanisms can contribute complex and interconnected mechanisms to the formation of pathological aggregates in ALS-FTD. Since the PQC system represents the first line of defense against aggregation, ongoing preclinical and clinical studies target different branches of the PQC and different steps of the aggregation process in an attempt to deliver efficient therapeutic strategies. Several clinical trials have been carried out in this direction already, holding promises for the treatment of ALS-FTD patients by targeting the overproduction of proteotoxic species and inhibiting the formation of protein aggregates [11]. One notable example is the use of colchicine, an alkaloid derived from the plant *Colchicum autumnale*, which has already received FDA approval for the treatment of other diseases [346]. Colchicine was shown to reduce the accumulation of TDP-43 misfolded species through the upregulation of HSPB8, which in turn promoted the clearance of DRiPs and other misfolded proteins [260,347]. Even though colchicine treatment was safe and showed some positive effects in the population tested, further investigations are required to assess its efficacy in larger cohorts [348].

Another promising strategy that has been developed involves the reduction in the upstream production of proteotoxic species by the use of antisense oligonucleotide (ASO) that are specific for the desired target mRNA [349]. Tofersen, as an example, is an FDA-approved treatment for ALS cases associated with SOD1 mutations; this ASO was indeed designed to target SOD1 mRNA, limiting the expression of the mutant protein without affecting the host DNA [350]. The same approach can be applied to other aggregation-prone proteins involved in ALS-FTD, such as ataxin 2, TIA-1, C9orf72 dipeptide repeats, and FUS, although it will be important to maintain adequate expression levels of the wild-type proteins that exert essential physiological functions [351,352,353,354].

Efforts should focus on a better understanding of how PTMs regulate the function and aggregation propensity of disease-linked proteins. Thanks to the advances in highly sensitive analytical techniques, we are uncovering novel PTMs in key ALS-FTD-associated proteins, underscoring the complexity and dynamic nature of protein aggregation in these disorders [240,242,274,299,318,338]. While some PTMs have historically been linked to the promotion of aggregation [243,245,247,307,355], others may even confer protective effects [249,286,310,356]. For instance, SUMOylation has emerged as a potential modifier of protein solubility, and it was shown to prevent TDP-43 aggregation under conditions of oxidative stress; conversely, impaired TDP-43 SUMOylation correlated with the development of TDP-43 pathology in an ALS mouse model [286,333]. Thus, identifying drugs and natural compounds that enhance the cellular defense mechanisms and target key PTMs that regulate protein stability and turnover may open new therapeutic avenues. Of note, SUMOylation activators, such as, for example, flavonoids, are under investigation for their potential neuroprotective properties against ALS and other neurodegenerative diseases associated with protein aggregation [357,358,359].

## Figures and Tables

**Table 1:** Main genes implicated in familial forms of ALS/FTD and their associated protein functions; u = undetermined. Adapted from Ragagnin et al., 2019 [6].

Gene	Protein	Locus	Frequency (%)	Putative Protein Function
*SOD1*	Superoxide dismutase 1	21q22.11	12–23.5	Scavenger enzyme, oxidative stress, UPS, autophagy
*ANG*	Angiogenin	14q11.1	1.5	Angiogenic factor
*SQSTM1/p62*	Sequestosome 1/p62	5q35	1.8	Autophagy
*DCTN1*	Dynactin 1	2p13.1	u	Axonal transport
*VAPB*	Vesicle-associated membrane protein (VAMP)-associated protein B	20q13.33	0.6	Vesicle trafficking, UPR
*VCP*	Valosin-containing protein	9p13.3	1–2.4	Autophagy
*TARDBP*	TAR DNA binding protein-43 (TDP-43)	1p36.22	5	DNA/RNA metabolism
*FUS*	Fused in sarcoma	16p11.2	5	DNA/RNA metabolism, stress granule function
*ATXN2*	Ataxin 2	12q24	5	RNA translation, exocytosis
*DAO*	D-amino acid oxidase	13q33.2	u	Oxidative deamination
*OPTN*	Optineurin	10p13	2.6	Autophagy
*C9orf72*	Chromosome 9 open reading frame 72	9p21.2	30–50 (Europe, North America)	Endosomal trafficking, autophagy
*TAF15*	TATA-binding associated factor	17q12	u	RNA metabolism
*UBQLN2*	Ubiquilin 2	Xp11.21	0.5–2.1	Autophagy, UPS
*PFN1*	Profilin-1	17p13	2.6	Actin dynamics
*hnRNPA1*	Human heterogeneous nuclear ribonucleoprotein A1	12q13.13	0.5	RNA metabolism
*hnRNPA2B1*	Human heterogeneous nuclear ribonucleoprotein A2B1	7p15.2	u	RNA metabolism
*CHCHD10*	Coiled-coil-helix-coiled-coil-helix domain containing 10	22q11.23	3.6	Mitochondrial function
*MATR3*	Matrin 3	5q31.2	1.8	RNA and DNA metabolism mRNA nuclear export
*TBK1*	TANK-binding kinase 1	12q14.2	1–5.2	Autophagy, inflammation
*TUBA4A*	Tubulin alpha-4A chain	2q35	1.1	Cytoskeleton
*SCFD1*	Sec1 family domain containing 1	14q12	u	Vesicle transport
*MOBP*	Myelin-Associated Oligodendrocyte Basic Protein	3p22.1	u	Compacting or stabilizing the myelin sheath
*C21orf2*	Chromosome 21 open reading frame 2	21q22.3	1.3–1.7	Ciliogenesis, DNA damage repair
*CCNF*	Cyclin F	16p13.3	0.6–3.3	UPS
*NEK1*	Never in mitosis gene A (NIMA)-related kinase 1	4q33	u	Cell cycle control and cilia regulation, DNA damage repair
*NEFH*	Neurofilament, heavy polypeptide 200kDa, heavy chain	22q12.1	1	Cytoskeleton

## Data Availability

No new data were created or analyzed in this study. Data sharing is not applicable to this article.
