# Correction: Verde et al. Molecular Mechanisms of Protein Aggregation in ALS-FTD: Focus on TDP-43 and Cellular Protective Responses. *Cells* 2025, *14*, 680

**DOI:** 10.3390/cells15121053

**Published:** 2026-06-09

**Authors:** Enza Maria Verde, Valentina Secco, Andrea Ghezzi, Jessica Mandrioli, Serena Carra

**Affiliations:** Department of Biomedical, Metabolic and Neural Sciences, University of Modena and Reggio Emilia, 41125 Modena, Italy; v.enzamaria96@gmail.com (E.M.V.); valentina.secco@unimore.it (V.S.); andreaghezzi140495@gmail.com (A.G.); jmandrio@unimore.it (J.M.)

In order to facilitate readers’ better understanding, some language descriptions and grammar as well as the layout of some chapters have been modified. 

## References

During manuscript preparation, a problem associated with Mendeley’s automatic reference numbering led to the incorrect placement of one citation (reference 198). With this correction, we revised the entire reference list leading to the following changes:

Previous reference 71 (Kim et al., 2017) was removed as it was not fully relevant to the content reported in the manuscript;Lopez-Erauskin et al., 2018 was added as reference 89 to provide additional support for the information reported in the manuscript;Previous reference 104 was substituted from Goutman et al., 2022 to Zhao et al., 2017 to improve coherence with the information presented;Previous ref 201 (Chiti et al., 2017) was removed as it was not fully relevant to the content reported in the manuscript;Previous reference 278 was updated, replacing Yewdell et al., 1996 with Schubert et al., 2000 to ensure greater consistency with the data discussed in the manuscript;The order of all references after 198 was adjusted accordingly due to the issue associated with Mendeley’s automatic reference numbering;Ref 181 was inserted in Section 2 as additional support for the information provided. TDP43 and Its Role in ALS-FTD, Section How TDP43 Properties Can Influence Its Behavior in ALS-FTD, Paragraph 6.

## Error in Figure

In the original publication [1], there was a mistake in Figure 2—as published. The sentence describing the effect of acetylation on TDP-43 was inadvertently omitted, while the sentence referring to TDP-43 SUMO2/3 conjugation was mistakenly repeated. The corrected Figure 2 appears below.

**Figure 2 cells-15-01053-f002:**
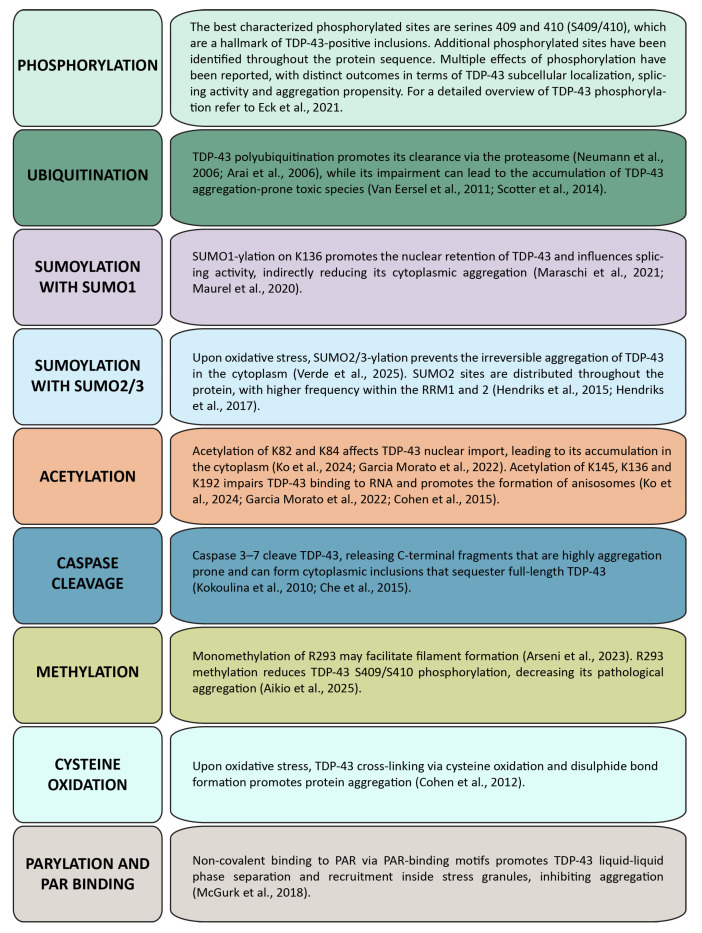
Known post-translational modifications of TDP-43 and their impact on protein function and aggregation-propensity [285,286,295–308].

The authors state that the scientific conclusions are unaffected. This correction was approved by the Academic Editor. The original publication has also been updated.
